# MYC and therapy resistance in cancer: risks and opportunities

**DOI:** 10.1002/1878-0261.13319

**Published:** 2022-10-20

**Authors:** Giulio Donati, Bruno Amati

**Affiliations:** ^1^ European Institute of Oncology (IEO) – IRCCS Milan Italy

**Keywords:** Myc, synthetic lethality, targeted therapy, therapy resistance

## Abstract

The MYC transcription factor, encoded by the c‐*MYC* proto‐oncogene, is activated by growth‐promoting signals, and is a key regulator of biosynthetic and metabolic pathways driving cell growth and proliferation. These same processes are deregulated in MYC‐driven tumors, where they become critical for cancer cell proliferation and survival. As other oncogenic insults, overexpressed MYC induces a series of cellular stresses (metabolic, oxidative, replicative, etc.) collectively known as oncogenic stress, which impact not only on tumor progression, but also on the response to therapy, with profound, multifaceted consequences on clinical outcome. On one hand, recent evidence uncovered a widespread role for *MYC* in therapy resistance in multiple cancer types, with either standard chemotherapeutic or targeted regimens. Reciprocally, oncogenic *MYC* imparts a series of molecular and metabolic dependencies to cells, thus giving rise to cancer‐specific vulnerabilities that may be exploited to obtain synthetic‐lethal interactions with novel anticancer drugs. Here we will review the current knowledge on the links between *MYC* and therapeutic responses, and will discuss possible strategies to overcome resistance through new, targeted interventions.

## Graphical abstract

Therapy resistance is a major limitation in clinical oncology. The MYC oncogene not only drives cancer progression, but also favors resistance to classical chemotherapy and targeted therapies. This review summarizes the clinical evidence and molecular mechanisms linking MYC and therapy resistance, and discusses possible innovative strategies to target the specific vulnerabilities of MYC‐driven cancer.

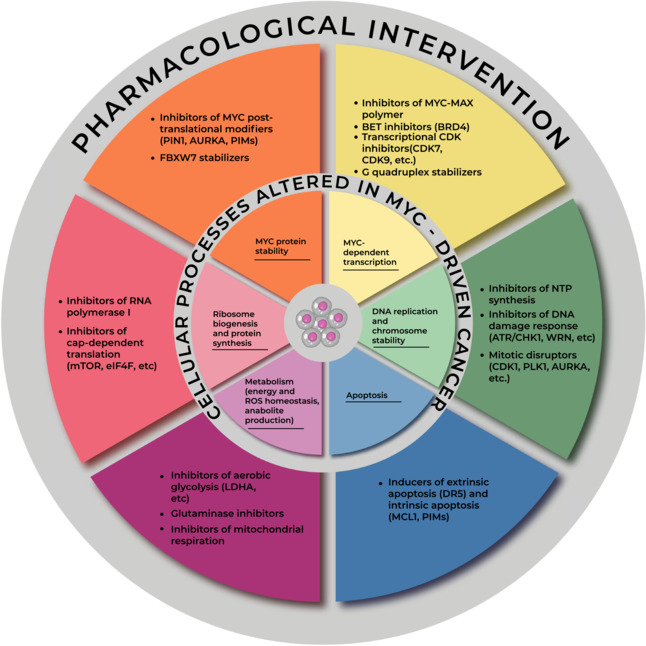

**Abbreviations**
5FU: 5‐fluorouracil
ABC: ATP‐binding cassette
ACT: adoptive cell therapies
AML: acute myeloid leukemia
ARK5: AMP‐activated protein kinase‐related kinase 5
ATR: ataxia telangiectasia and Rad3‐related
AURKA: Aurora A kinase
AURKB: Aurora B kinase
BCR: B‐cell receptor
BET: bromodomain and extraterminal domain
bHLH‐LZ: helix–loop–helix leucine‐zipper
CDK: cyclin‐dependent kinase
CHK1: checkpoint kinase 1
CLL: chronic lymphocytic leukemia
CML: chronic myeloid leukemia
CTP: cytidine triphosphate
CVD: cardiovascular diseases
DHL: double‐hit lymphomas
DLBCL: diffuse large B‐cell lymphoma
E‐box: enhancer‐box
EMT: epithelial‐to‐mesenchymal transition
ETC: electron transport chain
FBXW7: F‐box and WD repeat domain containing 7
G4: G‐quadruplex
ISR: integrated stress response
LDHA: lactate dehydrogenase A
MCL: mantle cell lymphoma
MM: multiple myeloma
MYC‐SL: synthetic‐lethal interaction with oncogenic MYC
OxPhos: oxidative phosphorylation
PIN1: peptidylprolyl cis/trans isomerase, NIMA‐interacting 1
PLK1: polo‐like kinase‐1
PP2A: protein phosphatase 2A
PRPS2: phosphoribosyl‐pyrophosphate synthetase 2
P‐TEFb: positive transcription elongation factor b
RNAi: RNA interference
ROS: reactive oxygen species
rRNA: ribosomal RNA
SAE: SUMO‐activating enzyme subunit
SCF: Skp, cullin, F‐box containing complex
SGOC: serine‐glycine‐one carbon
TCA: tricarboxylic acid
TNBC: triple‐negative breast cancers
UPR: unfolded protein response

## 1 Introduction

The c‐*MYC* proto‐oncogene (hereafter *MYC*) was identified over 40 years ago as the cellular homolog of the avian retroviral oncogene *v‐myc* [1, 2]. Structurally, MYC is part of a large class of transcription factors that contain a basic‐helix–loop–helix leucine‐zipper (bHLH‐LZ) motif, mediating dimerization and DNA binding [3]. MYC forms heterodimers with another bHLH‐LZ protein, MAX, allowing recognition of the so‐called Enhancer‐box (E‐box) consensus sequence CACGTG and variants thereof, primarily to promote or reinforce transcription [4, 5, 6]. Over three decades of research unraveled MYC's central role in cellular growth control, in normal physiology and development. Virtually every cell‐activating stimulus studied so far induces expression of the MYC transcription factor mRNA and protein product, which in turn coordinates complex gene expression programs involved in the many facets of cellular activation – ribosome and mitochondrial biogenesis, biosynthetic pathways, energy metabolism, cell growth, proliferation, and more [7, 8, 9, 10, 11, 12, 13]. This central position in the cell's regulatory circuitry endows *MYC* with high oncogenic potential, as its deregulated expression enforces the same cellular responses in an uncontrolled manner. Indeed, whether resulting from direct alterations of the locus (e.g., gene amplification, translocation) or from the activation of upstream signaling pathways (receptor tyrosine kinases, Ras, Raf, Wnt, Notch, etc.), most tumor types show deregulated *MYC* expression, resulting in uncontrolled activation of MYC‐driven programs. Altogether, overexpression of *MYC* – or of its paralogues, MYCN or MYCL – is a widespread event in most cancer types [7, 8, 9, 10, 11, 12, 13], and contributes to multiple hallmarks of the transformed phenotype [14, 15], including cell‐intrinsic and systemic features, such as angiogenesis, modulation of the tumor microenvironment, or immune evasion [13, 16].

While driving tumorigenesis, the overload of biosynthetic and metabolic activities activated by MYC elicits diverse forms of oncogenic stress, which impact on cancer initiation, progression and maintenance, as well as on the response to therapy. On one hand, oncogenic stress elicits a series of tumor‐suppressive responses (such as apoptosis, growth arrest, or senescence) [17, 18] that are normally bypassed during tumor evolution, but whose reactivation has emerged as a fundamental theme in cancer therapy – including treatment with classical chemotherapeutic agents (e.g., [19, 20, 21]). On the other hand, besides the above safeguard responses, oncogenic stress also elicits adaptive mechanisms that favor tumor cell survival and expansion, thus creating new dependencies – sometimes dubbed as ‘non‐oncogene’ addiction – that may also be targeted therapeutically [16, 22].

Altogether, the fitness of MYC‐overexpressing cells depends on a fragile equilibrium between ambivalent signals that not only elicit therapy resistance, but also provide new therapeutic opportunities. Here, we review the known links between *MYC* and either resistance or sensitization to therapeutic intervention, and draw future perspective to exploit these pharmaco‐genetic interactions toward improved cancer patient outcomes.

## 2 Cancer therapy: a brief historical perspective

The term cancer defines a large group of related, but heterogenous disorders characterized by abnormal cellular growth, which leads to invasion of surrounding tissues and, eventually, spreading to distant organs (metastasization). Among noncommunicable diseases, cancer is a major cause of premature death surpassed only by cardiovascular diseases (CVD). However, due to the increasingly aging population and better management of CVD, cancer is predicted to become the predominant cause of premature death by the end of the century [23].

Initially, the only treatment available to cancer patients was surgical resection of the tumor, with radiotherapy and chemotherapy becoming available during the course of the 20th century. Radiotherapy, which started to be used to treat cancer shortly after the discovery of X‐rays in 1895, is currently in use to treat superficial or localized neoplastic lesions, generally as part of multimodality treatments [24].

Chemotherapeutics are, in essence, drugs that kill proliferating cells and their preferential targeting of cancer cells is due to the latter undergoing unrestrained proliferation, which is one of the defining hallmarks of cancer [14]. The clinical use of chemotherapeutics started during the 1940s [25, 26], resulting in positive results that created great expectations in the oncology field. However, cancer cells soon proved to adapt to single agent therapies, with temporary remission shortly followed by disease relapse. To overcome these limitations, chemotherapy progressively evolved into a combination of anticancer agents (polychemotherapy) given at variable dose intensities in increasingly complex regimes, aimed at optimizing the therapeutic response while reducing toxicity to the patient.

Targeted therapy became the topic of intense studies prompted by the discovery of oncogenes and tumor suppressor genes in the 1970–1980s, the ever‐increasing understanding of the genetic determinants of cancer and, in recent years, by the surge of genome‐scale sequencing and other ‐omics technologies [27]. As a general principle, those molecular activities and signaling pathways that are altered in cancer cells which are required to sustain the main hallmarks of the transformed phenotype [14, 15] are obvious candidates for pharmacological intervention. We can trace the first concept of targeted therapy to the proposed removal of gonads to treat invasive breast cancer, made by Thomas Beatson at the end of the 19th century [28]. Over 40 years later, Charles Huggins formally made the connection between the shrinkage of sex hormone‐dependent breast and prostate cancer, with the removal of gonads and adrenal glands, as the source of these hormones [29]. Besides surgical removal of hormone‐producing glands, today's endocrine therapy of hormone‐dependent cancers includes pharmacological inhibition of hormone receptors or biosynthetic pathways [30].

Not considering endocrine therapy, the first drug specifically designed to inhibit an oncogenic pathway to successfully reach the clinic was imatinib, an inhibitor of the oncogenic tyrosine kinase BCR‐ABL1 [31]. Others followed suit, such as gefitinib and vemurafenib, which inhibit the oncogenic kinases produced by mutations in *EGFR* and *BRAF* (V600E) respectively [32, 33]. Targeted therapy also took advantage of synthetic‐lethal interactions prompted by the loss of specific tumor suppressor genes in cancer cells. For example, the PARP inhibitor olaparib proved effective against tumors characterized by loss of either BRCA1 or BRCA2 [34].

Even though cancer immunotherapy has only recently risen to fame, the first trials to induce inflammation to fight tumors date back to the end of the 19th century [35]. However, the origins of modern immuno‐oncology can be tracked to 1950s, with the concept of cancer immunosurveillance [36, 37]. Despite pioneering treatments to induce bladder tumor regression with attenuated bacteria [38], it was only in 1997 that the first immunotherapeutic drug, the monoclonal antibody rituximab, was approved to treat non‐Hodgkin's lymphoma [39]. Rituximab acts by binding CD20, a surface protein expressed in both mature and immature B‐cells, leading to their destruction by natural killer cells, and is currently used in combination with chemotherapeutic agents for the treatment of most lymphomas and leukemias of B‐cell origin [40] (see below).

Another monoclonal antibody, trastuzumab, was introduced in 1998 to treat breast cancer with high expression of the EGFR‐family receptor HER2 [41]. Indeed, the prognosis of this aggressive breast cancer subtype improved with trastuzumab, used either as single therapy or in combination with chemotherapy [42, 43]. More recently, harnessing the patient's immune system against the tumor through so called adoptive cell therapies (ACT) has been brought to the fore, and applied to cancers of different origin [44]. ACT entails the *in vitro* expansion of tumor‐specific cytotoxic T‐cells isolated from the tumor infiltrate before injecting them back in the patient. Alternatively, genetic engineering techniques have been applied to express T‐cell receptors against tumor antigens, thus extending the application range of ACT [45]. Finally, immune checkpoint inhibitors, such as anti‐PD‐L1 or anti‐CTLA4, are antibodies designed to block surface receptor–ligand interactions that suppress the immune response against cancer cells [46].

## 3 Therapy failure: one problem many faces

While continuous advances are being made in the clinical management of cancer, the improvement of patient survival – albeit with some notable exceptions – has been limited in the last decades [47]. The main reason hindering full cure is therapy resistance, or the unremitting capacity of cancer cells to effectively circumvent any new weapon that is thrown at them.

Whereas treatment‐resistant cancer cells may either pre‐exist or emerge during therapy, both scenarios are based on largely overlapping molecular and biological properties. In all cases, the main enabler of resistance is intra‐tumoral heterogeneity, a complex phenomenon that depends on an ensemble of cell‐intrinsic features, ranging from genetic and epigenetic diversity [48] to metabolic plasticity [49], and their interplay with cell‐extrinsic (i.e., environmental) variables [50]. Genetic heterogeneity arises from the relative instability of cancer genomes, compounded by specific mutation patterns derived from external factors (e.g., mutations induced by alkylating agents in cancers pretreated with temozolomide) [51]. Epigenetic heterogeneity is the by‐product of a wide range of alterations, such as DNA and histone methylation [52], resulting in phenotypically plastic and/or reversible differentiation programs (e.g., stem‐like properties, epithelial‐to‐mesenchymal transition, etc.) [53].

For a practical categorization of resistance mechanisms, we will hereby distinguish those that directly impact drug‐target interactions (on‐target) from off‐target mechanisms; the latter may act upstream or downstream of the target (pre‐ and post‐target, respectively) or may bypass its function altogether.

### 3.1 On‐target resistance

The effectiveness of a drug can be blunted by alterations in either the levels or the structure of its target. For example, shortly after the clinical introduction of the targeted drug imatinib, mutations in its receptor BCR‐ABL1 that hamper binding of the drug, were identified in relapsed chronic myeloid leukemia (CML) patients [54, 55]. Similar mechanisms are implicated in resistance to other targeted therapies, such as EGFR kinase inhibitors [56].

### 3.2 Off‐target resistance and bypass mechanisms

Targeted therapies exploit the addiction to mitogenic signaling gained through specific gain‐of‐function mutations. However, their effects can be lost due to the selection of clones where redundant pathways are activated, a mechanism known as ‘bypass resistance’. An example of bypass resistance is the activation of PI3K signaling by amplification of the *MET* oncogene in lung cancer resistant to EGFR inhibitor [57]. Reciprocally, activation of the EGFR pathway in MET‐addicted cancer cells confers resistance to MET inhibitors [58]. Similarly, resistance to inhibitors of BRAF V600E could be achieved either by alterations that bypass RAF‐dependent activation of the MAPK pathway [59], or by activation of alternative signaling pathway, such as PI3K [60].

Another off‐target mechanism lies in the acquisition of cellular phenotypes associated with resistance. In particular, reports linking the epithelial‐to‐mesenchymal transition (EMT) to chemoresistance have been steadily appearing since the 1990s [61]. Along this line, the potential for metastasization as the main pathologic feature of EMT has been put into question, in favor of inducing chemoresistance [62, 63]. Energy metabolism in cancer cells is highly plastic, allowing to adapt to variable external conditions [49]. Resistance to different classes of drugs have been causally linked to altered activity or expression of metabolic enzymes such as glycolytic, glutaminolytic or mitochondrial ones [64, 65]. Moreover, proficiency in the coupled mitochondrial processes of oxidative phosphorylation (OxPhos) and tricarboxylic acid (TCA) cycle is an absolute requirement for the intrinsically therapy‐resistant cancer stem cell phenotype in leukemia [66].

Finally, alterations in the tumor microenvironment might also provide cancer cells with the means to escape treatment. For example, secretion of the MET ligand hepatocyte growth factor by tumor stromal fibroblasts confers resistance to BRAF V600E inhibitors and correlates with poor prognosis in melanoma [67]. In an analogous manner, the efficacy of cytotoxic T‐cell based cancer immunotherapy can be reduced by immunosuppressive myeloid tumor infiltrates [68].

### 3.3 Pretarget resistance

Physical barriers that prevent a drug from reaching its intended target constitute pretarget mechanisms of resistance. Cancer burden has been inversely correlated with curability, owing not only to the increased probability to select for resistant cells [69], but also to inadequate blood flow creating a spatial gradient for chemotherapeutics, thus hindering their efficacy [70]. Effective drug exposure might also be precluded when cancer cells colonize ‘sanctuary sites’ in the body, the prototypical example being the central nervous system, where the presence of the blood–brain barrier prevents an effective exposure to systemically delivered drugs [71]. Alternatively, reduced drug concentration in neoplastic cells might follow from inactivating mutations in drug carriers or increased expression of proteins involved in drug efflux. The ATP‐binding cassette (ABC) family of membrane transporters are physiologically responsible for pumping out xenobiotics and endogenous metabolites, to prevent intracellular accumulation of these toxic moieties [72]. Neoplastic cells coopt this system by overexpressing ABCB1, also known as P‐glycoprotein, a promiscuous surface transporter that endows them with resistance to multiple, chemically distinct drugs [73, 74], a phenotype known as multidrug resistance.

### 3.4 Post‐target resistance

Cancer cells may mitigate and repair the damage caused by therapeutic agents, ultimately resulting in drug resistance and treatment failure. For example, the damage induced by genotoxic drugs and radiotherapy can be mitigated by enhanced mechanisms of DNA repair or scavenging of reactive oxygen species (ROS) [75, 76]. Refractoriness to cell death is another form of multidrug resistance, obtained by losing regulators and effectors of apoptosis and other forms of regulated cell death. For instance, loss of p53 or perturbed expression of BCL2‐family proteins, resulting in an impaired apoptotic response, are widely associated with resistance to different chemotherapeutics [77, 78].

## 4 MYC and therapy resistance

### 4.1 Clinical evidence

The *MYC* oncogene and its product not only exert a central role in cancer initiation and progression, but are also generally recognized as negative prognostic factors in diverse malignancies [79, 80, 81]. Here, we selectively focus on the available clinical data linking oncogenic MYC to therapy resistance in diverse cancer types (summarized in Table 1).

**Table 1:** MYC and therapy resistance in the clinic. The table provides the list of malignancies in which MYC alterations were documented to impact the response to the indicated therapies. IHC, immunohistochemistry; qNPA, quantitative nuclease protection assay; RNA‐seq, RNA sequencing; RT‐PCR, real‐time PCR.

Malignancy	Drug therapy	MYC alteration	References
Diffuse large B‐cell lymphoma	R‐CHOP immunochemotherapy	MYC translocation	[84, 85, 86, 87]
		MYC expression (IHC or qNPA; meta‐analysis)	[88]
		MYC/BCL2 co‐translocation and co‐expression (IHC)	[87, 89]
		MYC/BCL2 co‐expression (IHC)	[90]
		MYC/BCL2 co‐expression (IHC or qNPA; meta‐analysis)	[88]
Mantle cell lymphoma (MCL)	Ibrutinib targeted therapy	MYC gene signature (RNA‐Seq)	[101]
HER2‐positive breast cancer	Adjuvant chemotherapy (not specified)	MYC amplification	[102]
ER‐positive breast cancer	Adjuvant tamoxifen endocrine therapy	MYC gene signature (gene expression microarray)	[103]
	Endocrine therapy (not specified)	MYC amplification	[104]
Triple negative breast cancer	Neoadjuvant chemotherapy (not specified)	MYC gene signature (gene expression microarray)	[111]
		MYC/MCL1 co‐amplification	[110]
Colon cancer	Adjuvant 5‐fluoruracil chemotherapy	MYC expression (RT‐PCR)	[112]
	Anti‐EGFR targeted therapy + FOLFIRI chemotherapy	MYC expression (IHC)	[113]
Melanoma	Anti‐BRAF targeted therapy	MYC expression (IHC) and gene signature (gene expression microarray)	[114]

A tumor type that was extensively studied in this regard is diffuse large B‐cell lymphoma (DLBCL), the most common form of lymphoid malignancy in adults [82]. Currently, the front‐line therapy for DLBCL is R‐CHOP, a combination of the monoclonal antibody rituximab with four chemotherapeutic drugs (cyclophosphamide, doxorubicin and vincristine, and the glucocorticoid prednisone), which achieves cure in ca. 60% of patients. For relapsed and refractory DLBCL the success rates of salvage therapies are quite low [83]. *MYC* translocations and/or MYC protein overexpression are relatively frequent events in DLBCL (around 10% and 30% of cases, respectively) and, most importantly, have been linked to reduced patient survival [84, 85, 86, 87, 88]. DLCBL cases presenting high co‐expression of MYC and the anti‐apoptotic BCL2 protein show inferior prognosis [87, 88, 89, 90], which gets even worse in the subgroup historically known as ‘double hit lymphomas’ (DHL), a small subset (around 5% of total DLBCL) featuring concurrent chromosomal translocations targeting both *MYC* and *BCL2* [89, 91, 92].

The association between MYC and disease aggressiveness was documented in other B‐cell malignancies, including the progression of follicular lymphoma, mucosa‐associated lymphoid tissue lymphoma, and chronic lymphocytic leukemia (CLL) from their indolent forms to more aggressive, treatment‐refractory phases [93, 94]. In particular, recent multi‐omic studies in CLL identified MYC activity as one of the main features associated with morbidity, either within the chronic phase [95] or during the evolution to high‐grade lymphoma [96] (a process known as Richter transformation).

Mantle cell lymphoma (MCL) is a largely incurable B‐cell malignancy [97] that heavily relies on B‐cell receptor (BCR) signaling for survival and propagation [98]. Targeting Bruton's tyrosine kinase, an essential mediator of BCR signaling, with the covalent inhibitor ibrutinib proved effective to treat relapsed and refractory MCL [99]. However, resistance to ibrutinib monotherapy inevitably emerges, with resistant MCL also showing poor response to salvage chemotherapies [100]. Comparing mRNA profiles from clinical specimens of ibrutinib‐resistant and sensitive MCL led to the identification of several resistance‐associated signatures, with a MYC‐driven transcriptional program being the most significantly enriched [101]. Most noteworthy here, other enriched signatures included OxPhos and mTOR [101], which were also linked to disease aggressiveness and Richter transformation in CLL [95, 96]. The connections between MYC and either of these features, as well as their therapeutic implications, will be examined more in detail below.

A number of observations also linked MYC to therapy resistance in solid tumors. In HER2‐positive breast cancers, for example, amplification of the *MYC* locus identified a subgroup of patients with particularly poor prognosis when treated with adjuvant chemotherapy [102]. Likewise, high expression of the MYC protein or a MYC‐dependent gene signature predicted poor prognosis in patients suffering from estrogen receptor‐positive breast cancer treated with adjuvant hormonal therapy [103]. Accordingly, compared to before treatment, tumoral tissue from patients who relapsed after endocrine therapy showed *MYC* amplification, [104]. MYC activity and/or expression are also upregulated in triple‐negative breast cancers (TNBC) [105, 106, 107], a subtype characterized by lack of estrogen, progesterone and HER2 receptors, and associated with higher risk of recurrence and death [108, 109]. In a subset of TNBC cases, *MYC* was co‐amplified with *MCL1* (an anti‐apoptotic member of the BCL2‐family), an occurrence further enriched in residual disease after neoadjuvant therapy, suggesting a role in chemoresistance [110]. Finally, another study confirmed that a MYC‐driven gene signature correlated with TNBC status, and was even a better predictor of disease outcome in breast cancer patients [111].

In colon cancer, high expression of the MYC transcript significantly correlated with tumor recurrence in patients who underwent adjuvant 5‐fluorouracil (5FU) chemotherapy, an association attributed to the MYC‐dependent activation of the ABC‐family transporter ABCB5 [112]. High MYC protein expression in primary colon cancer was also predictive of an inferior response to anti‐EGFR monoclonal antibodies plus FOLFIRI (a polychemotherapy regimen based on 5FU and irinotecan) [113]. The same study found MYC more expressed in metastases resected during the course of therapy or during the resistance phase, as compared to naïve primary tumors [113]. High MYC expression in BRAF‐mutant melanomas that progressed after BRAF inhibitor therapy was identified as the common denominator between diverse resistance pathways (ERK, PI3K, etc.) [114]. Most importantly, while MYC overexpression induced resistance to BRAF inhibitors in melanoma cells *in vitro*, it also sensitized the cells to inhibitors of glucose metabolism, glutaminolysis and other metabolic processes, pointing to actionable MYC‐induced metabolic dependencies [114]. The concept of synthetic‐lethal interactions between oncogenic MYC and pharmacological inhibition of distinct pathways will be discussed below (see Section 4.3).

Albeit conclusive clinical evidence linking MYC activity to immunotherapy response is still lacking, a strong body of preclinical studies unraveled a prominent role of oncogenic MYC in evading immune surveillance, in particular by promoting the expression of surface receptors and cytokines (e.g., PD‐L1 and CCL9, respectively) that establish immune tolerance in the tumor microenvironment [16]. Indeed, de‐activating the oncogene in various MYC‐driven mouse tumor models prompted systemic tumor regression with – among other effects – marked reactivation of anti‐tumoral immune responses [115, 116, 117]. Likewise, suppressing MYC via epigenetic therapy reverted immune evasion in a mouse lung cancer model [118]. Finally, a retrospective analysis of several clinical studies suggested that elevated MYC expression might be associated with resistance to immune checkpoint inhibitor therapy in metastatic urothelial carcinoma and possibly other cancer types, including TNBC [119]. While the significance of these associations remains to be confirmed, the same study reported that MYC‐induced anti‐PD‐L1 resistance could be overcome with a combinatorial immuno‐therapeutic regimen in a preclinical model of TNBC. Altogether, these observations warrant further studies on the mechanisms linking MYC to immunotherapy resistance, and on the best means to counteract them therapeutically.

### 4.2 All roads lead to Rome: strategies to target MYC in cancer

Inactivating MYC is sufficient to induce cancer regression in diverse models of MYC‐driven cancer lymphoma, skin papilloma, and osteosarcoma, a phenomenon known as ‘oncogene addiction’ [16, 22, 120, 121, 122, 123, 124]. Thus, targeting MYC activity seems to be a promising strategy to treat MYC‐driven, and possibly other types of cancer [117, 125, 126]. However, as for other transcription factors, several characteristics of the MYC protein, such as the lack of a catalytic cleft and nuclear localization, limit effective targeting by either small molecules or antibodies. The fact that no targeted therapy against MYC has been approved for clinical use so far, made this oncogene a prime example of attractive, yet ‘undruggable’ target [127]. Hereafter, we summarize the different strategies that have been tested to solve the challenge posed by MYC, with a focus on those that already led to clinical studies; for a more systematic coverage, we refer the reader to dedicated reviews on the subject [128, 129, 130, 131].

A first attempt to down‐regulate MYC expression for therapeutic means stemmed from the identification of a guanine‐rich region that could organize itself in a higher‐order DNA structure known as G‐quadruplex (G4) within the human *MYC* promoter. Stabilization of the G4 structure with small molecules led to transcriptional silencing of the *MYC* gene [132]. In subsequent years, two molecules targeting the *MYC* G4 structure were tested in the clinic: quarfloxin/CX‐3543 and APTO‐253. The former, while initially selected for its binding to the MYC G4, was later shown to disrupt the binding of nucleolin to nucleolar G4s, thus inhibiting ribosomal RNA (rRNA) transcription [133]. Its clinical development ceased after completion of a phase II study in neuroendocrine tumor patients in 2011 (https://ClinicalTrials.gov/show/NCT00780663). The G4 stabilizer APTO‐253 showed the ability to repress MYC in acute myeloid leukemia (AML) cells [134] and was tested in a phase I clinical trial in patients with relapsed AML (https://ClinicalTrials.gov/show/NCT02267863). However, the study was terminated and further drug development abandoned (https://www.aptose.com/news‐media/press‐releases/detail/220/aptose‐provides‐update‐on‐apto‐253‐program). Despite these setbacks, the development of G4 stabilizers designed to repress MYC and other clinically relevant targets remains the focus of continued efforts [130, 135].

Another strategy employed to directly suppress MYC activity is to hamper dimerization with its obligate partner MAX or the subsequent binding to E‐box consensus elements in genomic DNA, both of which are essential for the transcriptional and transforming activities of MYC [5, 6, 136]. The search and identification of molecules able to interfere with these processes have been the scope of intense efforts in numerous laboratories [128, 129, 130, 131]. Of note here, several of these molecules displayed reasonable *in vivo* efficacy and tolerability profiles in preclinical studies [137, 138, 139, 140, 141, 142]. In most instances, however, the range of off‐target effects and mechanisms of action of these molecules remain to be addressed.

Years before any other MYC:MAX inhibitor, a 90‐residue peptide spanning the bHLH‐LZ dimerization domain of MYC with targeted amino acid substitutions, termed Omomyc, was shown to bind MYC, sequester it away from MAX, and suppress cell proliferation [143]. In murine models, transgenic expression of Omomyc prevented MYC‐driven skin tumorigenesis and induced regression of Ras‐driven lung and pancreatic adenocarcinomas [117, 125, 126, 144]. Finally, the Omomyc peptide was shown to be cell‐permeable, distribute widely in the body and exert an effective anti‐tumoral activity against Ras‐induced lung cancer upon intranasal administration in mice [145]. Based on its efficacy in these preclinical studies, Omomyc is currently being tested in a phase I/II hybrid clinical trial to assess safety and efficacy in patients with solid tumors (https://ClinicalTrials.gov/show/NCT04808362).

Besides the aforementioned efforts to target MYC directly, multiple laboratories pursued alternative strategies to suppress MYC activity, in particular by targeting factors regulating MYC degradation. The Aurora A kinase (AURKA), known for regulating a mitotic cell cycle checkpoint [146], was later shown to be able to bind to MYC and prevent its ubiquitination and degradation [147]. Concurrently, the expression of *AURKA* and of the paralog *AURKB* – also involved in the control of mitosis [148] – is positively regulated by oncogenic MYC [149], thus establishing a positive feedback among these oncogenes. Indeed, in a murine model, pharmacological inhibition of Aurora kinases proved highly effective against MYC‐driven lymphoma [149]. In line with these findings, the specific AURKA inhibitor MLN8237 (alisertib) facilitated degradation of either MYC or its paralog MYCN by the FBXW7‐associated ubiquitin‐ligase complex SCF^FBXW7^, and induced tumor regression in preclinical studies [147, 150]. Among AURKA inhibitors, alisertib was the most extensively tested in the clinic, and yielded promising results in monotherapy, despite toxicity‐related concerns [151]. However, it later failed to show improvements over other single‐agent therapies in a phase III trial on relapsed/refractory T‐cell lymphoma [152]. Finally, given the role of AURKA in the mitotic checkpoint, it is worth reminding that oncogenic MYC also sensitizes cells to mitotic disruptors [153] and thus, alisertib may exert its synthetic‐lethal interaction with MYC by the same mechanisms described below for this class of drugs.

Other potential targets involved in the control of MYC stability are the peptidylprolyl cis/trans isomerase PIN1 and the protein phosphatase PP2A. PIN1 induces a conformational change in pS62/pT58 MYC that allows the binding by PP2A, which in turn dephosphorylates pS62 to facilitate MYC degradation by the SCF^FBXW7^ complex [154]. PIN1 is involved in the development of various cancers and has thus attracted attention as a possible target for cancer therapy, with several PIN1 inhibitors described and tested in preclinical studies [155]. While the clinical properties of these inhibitors remain to be assessed, it was reported that All‐*trans* retinoic acid, a drug used to treat promyelocytic leukemia expressing the PML‐RARα fusion protein, directly binds and inhibits PIN1, which may extend its use to other cancer types [156]. PP2A has a very broad range of substrates, with specificity given by the subunit composition of the holoenzyme, and generally act as a tumor suppressor [157, 158]: for this reason a more sensible approaches for cancer treatment could be either to use pharmacological activators of specific PP2A isoforms [159, 160, 161], or to target its cellular inhibitors. The latter is best exemplified by CIP2A, an endogenous inhibitor of PP2A able to stabilize MYC and often found overexpressed in cancer [162]. While no modulator of PP2A activity has been tested in the clinic as yet, the clinically approved drugs bortezomib and erlotinib, a proteasome and an EGFR inhibitor respectively, showed off‐target inhibitory activity on CIP2A [158].

Polo‐like kinase‐1 (PLK1) phosphorylates FBXW7, promoting its auto‐ubiquination and degradation, thus stabilizing both MYC and MYCN [163, 164]. Furthermore, inhibition of PLK1 with BI6727 (volasertib) synergized with the BCL2 inhibitor ABT199 (venetoclax) to kill MYC/BCL2 double‐hit lymphoma cell lines [164]. The clinical development of volasertib was discontinued in 2018 due to failure of reaching the primary endpoint in a phase III study in AML patients [165], but should resume following a licensing agreement with a new developer (https://www.nfcr.org/blog/ricardo‐garcia‐the‐power‐of‐repurposing/).

A different approach tested to indirectly target MYC was to block the interaction between acetylated histones and the bromodomain protein BRD4. BRD4 and the other three members of the bromodomain and extraterminal domain (BET) family recognize acetylated histones and facilitate transcription by recruiting the positive transcription elongation factor b (P‐TEFb) to target loci, which include *MYC* itself [166]. Moreover, both BRD4 and P‐TEFb, including its catalytic subunit CDK9 (see below), associate with MYC and contribute to its transcriptional activity [167, 168, 169]. JQ1, a small molecule inhibitor of BET proteins, suppressed *MYC* expression in preclinical models of multiple myeloma (MM) and AML, effectively halting cancer growth *in vitro* and *in vivo* [170, 171], although these and other studies indicated that BRD4 inhibition has multiple consequences beyond MYC inhibition [172, 173, 174].

Derivatives of JQ1 and other BET inhibitors tested in phase I/II clinical trials on both hematological and solid tumors patients have shown promising results in terms of efficacy, but also raised concerns regarding their safety due to frequent thrombocytopenia and other adverse events [175]. Currently, there is an ongoing phase III trial with the BET inhibitor CPI‐0610 (pelabresib), reported to inhibit MYC [176], in combination with the JAK inhibitor ruxolitinib to treat myelofibrosis (https://ClinicalTrials.gov/show/NCT04603495).

### 4.3 Synthetic lethality: exploiting oncogenic pathways for their own demise

**Fig. 1:**
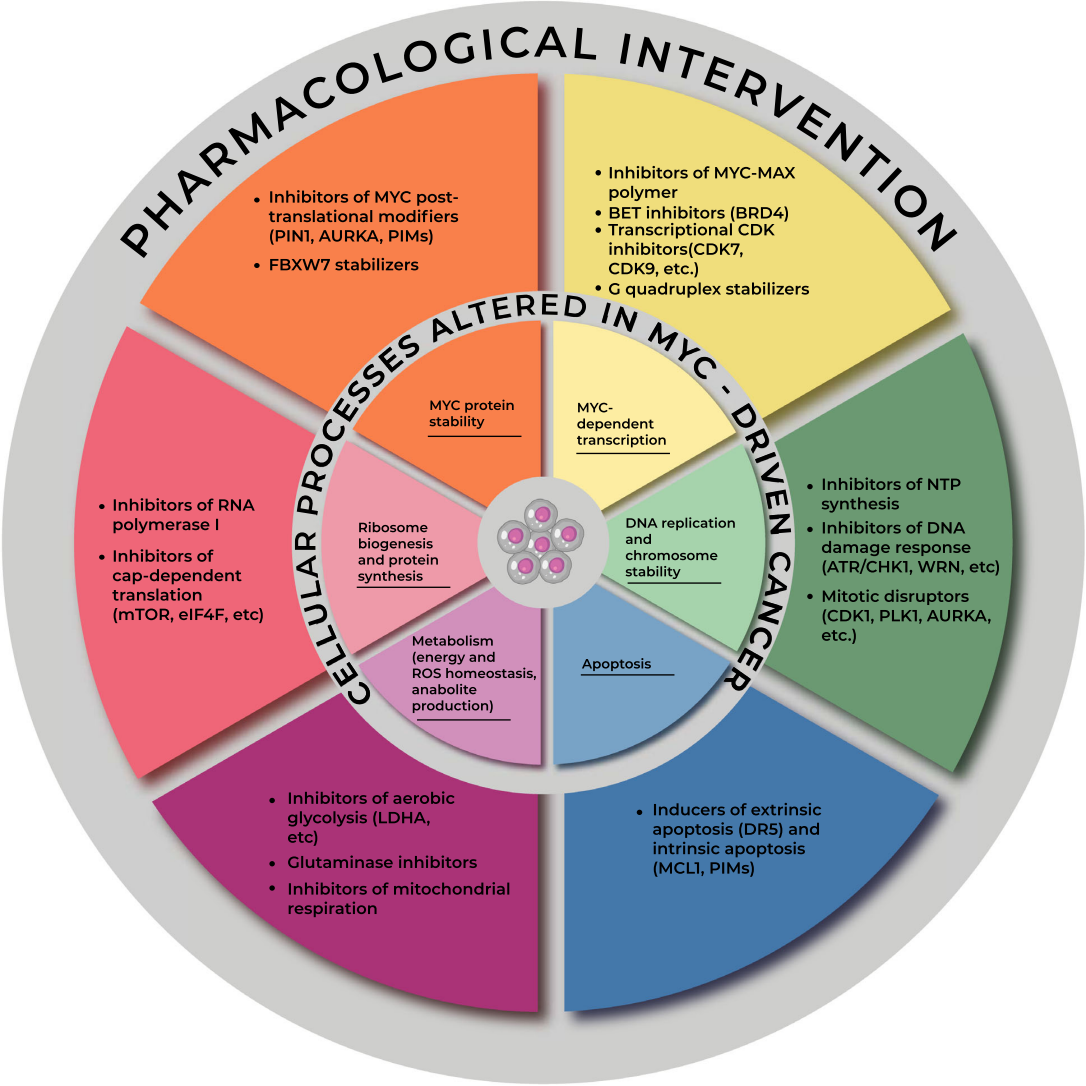
Tackling oncogenic MYC. The MYC transcription factor regulates diverse cellular processes (inner circle) that promote cell growth and proliferation, and are hijacked in cancer cells to fuel tumor aggressiveness and/or therapy resistance. However, the same processes may become a dependency in MYC‐driven cancer, and may thus be exploited for pharmacological intervention (outer circle): drug classes that impact these processes are indicated with their relevant molecular targets.

Like its physiological counterpart, oncogenic MYC promotes energy production and anabolic pathways, but does so unceasingly, regardless of external growth signals, to sustain cancer hyperproliferation [11, 12, 13]. Therefore, the associated metabolic reprogramming creates multiple dependencies that can be therapeutically exploited [177, 178]. Moreover, due to their altered biology, cancer cells are exposed to a variety of endogenous stresses, such as DNA damage/replication, mitotic, metabolic, oxidative and proteotoxic stress. These so called ‘stress phenotypes’ (all of which have all also been connected to oncogenic MYC; e.g., [179, 180, 181, 182]) are proposed to be an integral part of neoplastic characteristics, and interfering with pathways required to mitigate their effects represents a valid therapeutic strategy [22]. How the aforementioned MYC‐dependent processes may be exploited therapeutically is schematically illustrated in Fig. 1 and will be discussed in the next two sections.

The identification of druggable targets for a synthetic‐lethal interaction with oncogenic MYC (MYC‐SL) has been pursued either in a hypothesis‐based manner, based on the knowledge of pathophysiological changes induced by the oncogene, or by large‐scale screens using either custom‐built RNA interference (RNAi) or drug libraries [183, 184]. As an example of the latter approach, an RNAi library targeting ‘druggable’ gene products was used to screen for MYC‐SL interactions in non‐transformed fibroblasts modified to over‐express MYC [185], identifying 40 MYC‐SL potential targets. Among the candidates from this screen, the chromatin regulator CECR2 was recently shown to play a role in promoting breast cancer metastasization [186]. In another study, based on the known induction of intrinsic apoptosis by oncogenic MYC, the authors tested which anti‐apoptotic BCL2‐family protein would be required for survival of MYC‐driven lymphomas, uncovering a critical role for MCL1, independently of the cell's p53 status [187]. MCL1 is an attractive therapeutic target, being frequently overexpressed and associated with poor prognosis in cancer, with several MCL1 inhibitors currently in phase I/II clinical studies [188]. Besides the intrinsic apoptotic pathway, controlled by BCL2‐family proteins and triggered by permeabilization of the outer mitochondrial membrane and consequent release of cytochrome c, apoptosis can be induced through the extrinsic pathway, activated by death receptors of the TNF superfamily [189]. In another study, ligands for several death receptors were tested in MYC‐overexpressing cells, leading to the identification of the DR5 ligand, TRAIL, as a critical MYC‐SL interactor [190].

The PIM family is constituted of three homologous serine/threonine kinases that are frequently over‐expressed in diverse human cancers [191]. In murine tumor models, PIM kinases are weak oncogenes by themselves, but can exert strong cooperation with other oncogenes, in particular with MYC and MYCN in lymphomagenesis and prostate carcinogenesis [192, 193, 194, 195]. The basis of this oncogenic cooperation has been ascribed to phosphorylation of several substrates, including (a) MYC itself, thus increasing its stability [196], (b) histones at a subset of MYC target genes, facilitating MYC‐dependent transcription [197], and (c) the BCL2‐family protein BAD, hampering its pro‐apoptotic activity [198, 199]. Furthermore, the three PIM‐family kinases proved redundant for the cooperation with oncogenic MYC [200]. Several small molecule inhibitors of PIM kinases are currently in clinical development [191, 201] and some of them showed synthetic lethality with MYC and synergy with anti‐CD20 immunotherapy in preclinical models of MYC‐driven TNBC and DLBCL, respectively [202, 203].

Replication stress and genomic instability are part of the cancer‐associated phenotypes [15] and are found in MYC‐driven cancer [181, 182]. Since the ATR‐CHK1 branch of the DNA damage response mitigates genome instability in cancer cells [204], inhibiting this signaling axis has the potential for a MYC‐SL. Indeed, CHK1 inhibitors proved effective in killing murine MYC‐driven lymphoma [205]. Similarly, an RNAi screening identified CHK1 depletion as synthetic lethal with MYCN overexpression in neuroblastoma cells [206]. Both ATR and CHK1 inhibitors have reached the clinical trial stage, with encouraging results especially for ATR inhibitors [207]. Two phase II studies, in particular, proved the effectiveness of combining the ATR inhibitor berzosertib with chemotherapeutics to treat platinum‐resistant high‐grade serous ovarian cancer and lung small cell neuroendocrine cancers [208, 209], two aggressive forms of neoplasia bearing frequent amplification of MYC family members [210, 211, 212].

The *WRN* gene, which encodes for a protein with helicase and exonuclease activity involved in genomic integrity, is mutated in Werner syndrome, a form of progeria whose features include early onset cancer [213]. While WRN is commonly regarded as a tumor suppressor, its pharmacological inhibition was proposed to achieve synthetic‐lethal effects in neoplastic cells characterized by high replication stress [214, 215], which may render it effective also against MYC‐overexpressing cancer. This assumption is supported by preclinical evidences showing that the *WRN* locus is a direct target of MYC [216], and that its deficiency hampers MYC‐driven lymphomagenesis in mice [217].

Targeting mitotic processes is also a sensible choice to pursue synthetic lethality with MYC, since this oncogene has been associated with chromosomal instability [218, 219, 220]. Indeed, MYC overexpression, while activating the expression of mitotic spindle genes [221, 222], also facilitates cell death when these genes are selectively depleted by mRNA knockdown [223] or when cells are treated with mitosis disruptors (e.g., AURKA inhibitors) [153]. The MYC‐dependent sensitization to mitotic perturbations was also confirmed by an unbiased RNAi screen, which found that depleting SUMO‐activating enzyme subunits 1 or 2 (SAE1/2), both required for protein SUMOylation [224], was synthetical lethal with MYC [225]. In this context, SAE2 depletion modulated the expression of a group of mitotic spindle genes, which switched from being MYC‐induced to MYC‐repressed, thus causing chromosomal abnormalities and growth‐arrest in MYC‐overexpressing cells [225].

Members of the Cyclin‐dependent kinase (CDK) family control progression through the different phases of the cell cycle: CDK1, in particular, is essential for entry and progression through the mitotic phase [226]. Pharmacological inhibition of CDK1 led to selective killing of cells expressing oncogenic levels of MYC and reduced *in vivo* growth of MYC‐driven lymphomas [227]. The specificity of the synthetic‐lethal interaction between MYC and CDK1 was validated using a temperature‐sensitive CDK1 mutant cell line [227], as well as through pharmacological inhibition in cells lacking other CDKs [228]. CDK2 controls entry and progression through the S phase [226]. Its pharmacological inhibition revealed a noncanonical role in preventing MYC‐driven senescence [229] and mediating the cooperation between MYC and RAS oncogenes [230].

Apart from the cell cycle‐regulatory CDKs, other members of this kinase family control RNA transcription [231, 232]. CDK7 and CDK9 in particular, the catalytic subunits of the TFIIH and P‐TEFb complexes, phosphorylate distinct serine residues in the RNA polymerase II C‐terminal domain, favoring the orderly succession of transcriptional initiation and pause‐release, respectively [233]. An RNAi screen among known drug targets pointed to CDK9 as a critical activity for survival of MYC‐overexpressing/p53‐deleted murine hepatocellular carcinoma cells [234]. Moreover, the authors found that enforcing MYC expression in human hepatocellular carcinoma‐derived cells increased the sensitivity to PHA‐767491 [234], a CDK9‐inhibitory tool compound [235]. Similarly, the CDK9 inhibitor AZ5576 efficiently targeted DLBCL cells expressing high MYC level *in vitro* and *in vivo*, and its effects were potentiated by enforcing MYC expression [236]. Based on these results, AZ5576 has been tested in a phase I clinical study on patients with relapsed or refractory hematological malignancies (https://ClinicalTrials.gov/show/NCT03263637).

CDK7 might also be an attractive target in MYC‐driven tumors: indeed, the covalent CDK7 inhibitor THZ1 [237] disproportionally repressed super‐enhancer regulated genes, including MYC, MYCN and MYCL in diverse cancer‐derived cell lines [238, 239]. Due to the high homology between CDK‐family kinases, small‐molecule inhibitors often target multiple members. For example, THZ1 also inhibits the transcriptional regulators CDK12 and CDK13, and combined inhibition of CDK7/12/13 was required to suppress MYC expression in ovarian cancers harboring MYC amplification [210]. Hence, while conveying the risk of increased toxicities, multi‐target inhibitors might also have improved clinical potential. Along the same line, an *in vivo* efficacy and tolerability screen for clinically suitable CDK inhibitors pointed out to dinaciclib, a composite CDK1/2/5/9 inhibitor, as an effective anti‐cancer agent [240]. In preclinical studies, dinaciclib showed strong antitumoral activity against breast cancer and B‐cell lymphoma expressing high levels of MYC [111, 241]. Finally, in contrast with the aforementioned synthetic‐lethal interactions, it is noteworthy here that elevated MYC activity may also induce resistance to inhibitors of CDK4 and CDK6 [242, 243]. Hence, while the list of clinically relevant CDK inhibitors is steadily growing [244], whether MYC expression and/or activity may be used to predict the response of cancer cells to these molecules remains an important open question that needs to be systematically addressed.

To sustain increased rates of DNA replication and RNA transcription, oncogenic MYC promotes nucleotide biosynthesis [13, 245]. Phosphoribosyl‐pyrophosphate synthetase 2 (PRPS2) catalyzes the first step of *de novo* nucleotide biosynthesis [246]. PRPS2 expression was promoted by MYC at the transcriptional, as well as at the translational levels and, most importantly, was rate‐limiting for MYC‐driven lymphomagenesis [247]. Other enzymes in the same pathway were also induced by MYC and one of these, ADSL, was identified through an *in vivo* RNAi screen as a critical MYC effector in lymphoma [248]. Another metabolic process whose components are often up‐regulated in cancer is the serine‐glycine‐one carbon (SGOC) pathway, which controls purine and dTMP biosynthesis [249]. Most relevant here, up‐regulation of the SGOC pathway correlated with MYCN amplification in neuroblastoma, and MYCN sensitized neuroblastoma cells to pharmacological inhibition of one of its components, phosphoglycerate dehydrogenase [250].

Cytidine triphosphate (CTP) is the least abundant among the four nucleotides [246]. Pharmacological inhibition of CTP synthase induces selective replication stress in MYC‐overexpressing cancer cells and synergizes with ATR inhibitors to kill them [251]. Cell lines derived from relapsed small‐cell lung cancer presented higher MYC mRNA levels and/or more frequent amplification of MYC‐family genes than those derived from treatment‐naïve patients, showed higher expression of genes involved in purine synthesis, and were particularly sensitive to the immunosuppressant mizoribine [252], which inhibits inosine monophosphate dehydrogenase, the rate‐limiting enzyme of guanine biosynthesis [253]. Interestingly, the consequent depletion of guanine hampered the activity of cellular GTPases required for RNA polymerase I recruitment onto ribosomal DNA [252]. This was most likely responsible for the observed MYC‐SL effect of mizoribine, since rRNA transcription is rate limiting for ribosome biogenesis [254]. Indeed, activation of genes involved in ribosome biogenesis and protein synthesis are among the most conserved activities of MYC, consistent with its central role in cell growth and proliferation [255], and murine MYC‐driven lymphomas proved to be highly sensitive to the impairment in protein synthesis consequent to reduced ribosome biogenesis, either by knocking out a ribosomal protein [256] or by pharmacological inhibition of RNA Polymerase I – and thus of rRNA synthesis [257]. Similarly, direct inhibition of protein translation by knockdown or pharmacological inhibition of the eIF4F complex was synthetic‐lethal with oncogenic MYC in murine models of lymphoma and myeloma [258, 259].

mTOR kinase activity is regulated by nutrient levels and mitogenic cues converging on the PI3K/AKT pathway [260], and downstream targets of mTOR (e.g., S6K1, 4E‐BPs, etc.) promote ribosome biogenesis and cap‐dependent protein translation [261]. Oncogenic MYC promotes mTOR activation by increasing essential amino acid import [262], while mTOR signaling positively regulates MYC protein translation and stability [263, 264, 265, 266], thus creating a positive interplay among these crucial regulators of cell growth. In line with these findings, pharmacological inhibition of mTOR/PI3K exerted anti‐cancer effects and decreased MYC levels in preclinical models of breast cancer, MM and AML [267, 268, 269], and suppressed the expression of MYC targets in CLL cells [95]. In a transgenic model of MYC‐driven lymphoma, tumor initiation and maintenance were hampered by the mTOR inhibitor everolimus, albeit without suppressing MYC level and activity [270]. In apparent contrast, increased MYC expression was reported to confer resistance to everolimus [271]. The rapalogs (i.e., mTOR allosteric inhibitors) everolimus and temsirolimus are the only mTOR inhibitors clinically approved so far for the treatment of several solid tumors, but showed rather limited efficacy both as monotherapy and in combination [272]. The limitations of rapalogs as therapeutic agents and the structural similarities between mTOR and PI3K pushed the development of dual PI3K/mTOR inhibitors, several of which reached the clinical research stage [272]. One of these drugs, BEZ235 (dactolisib), showed efficacy on preclinical models of MYC‐driven lymphoma, mediated by inhibition of the DNA damage response kinase ATM along with mTOR [273]. Nonetheless, as with everolimus, others reported that MYC overexpression might be linked to resistance to dual PI3K/mTOR inhibitors [274, 275]. Whether this class of drugs would be an effective treatment for MYC‐driven cancer remains to be systematically addressed.

Therapeutically relevant MYC‐SL interactors have also been identified among enzymes and regulators of energy metabolism. For example, an RNAi screen focused on the human kinome showed that depletion of ARK5 led to cell death in the presence of high MYC activity. This was due to simultaneous high rates of protein synthesis and reduced OxPhos activity, thus leading to disruption of energy homeostasis [276]. In a model of MYC‐driven liver cancer, upregulation of lactate dehydrogenase A (LDHA) and LDHA‐dependent aerobic glycolysis was associated with the acquisition of a fully transformed phenotype [277]. Reciprocally, pharmacological inhibition of LDHA or nicotinamide phosphoribosyl‐transferase, both required for proficient aerobic glycolysis, led to selective toxicity toward MYC‐overexpressing pancreatic cancer and glioblastoma cells [278, 279].

Reliance on glutamine to fuel the mitochondrial TCA cycle is one of the best characterized metabolic alterations in cancer cells [280]. Oncogenic MYC promotes glutamine uptake and glutaminolysis, creating an addiction to glutamine and sensitizing cancer cells to glutaminolysis inhibitors [281, 282]. In MYCN amplified neuroblastoma, glutamine deprivation induces the expression of pro‐apoptotic BCL2‐family proteins and subsequent cell death, dependent upon the transcription factor ATF4 [283], the main effector of the Integrated Stress Response (ISR) [284, 285]. A more detailed discussion on the role of the ISR in the selective killing of MYC‐overexpressing will be provided below.

### 4.4 The case of OxPhos inhibitors to treat MYC‐driven cancer

Following from the profiling of genes up‐regulated in MYC‐driven lymphomagenesis [221], our laboratory undertook an *in vivo* RNAi screen aimed at the identification of critical MYC effectors [248]. Among other candidates, this pointed to the mitochondrial ribosome as an essential mediator in lymphoma maintenance. Tigecycline is a clinically approved antibiotic that inhibits not only bacterial translation [286], but also mitochondrial translation, with consequent impairment of OxPhos activity [287]. Hence, we hypothesized that this drug could be used to exploit the MYC‐induced dependency upon mitochondrial translation. Indeed, we and others showed that tigecycline was synthetic‐lethal with MYC overexpression in cultured B‐cells, and killed MYC‐driven B‐cell lymphomas [248, 288]. Following up from these results, we showed that tigecycline and venetoclax acted synergistically against tumor xenografts derived from MYC/BCL2 double‐hit lymphoma [289]. Hence, targeting the mitochondrial ribosome – and ultimately OxPhos activity – provided relevant therapeutic leverage against aggressive MYC‐associated lymphoma.

Promoting mitochondrial biogenesis is an important contribution of MYC to normal cell physiology [290], which would be fitting with the dependency for mitochondrial translation induced by oncogenic MYC. In fact, oncogenic MYC increases the reliance upon mitochondrial metabolism in B‐cell lymphoma, as assessed in a cellular model of conditional MYC repression [291]. The potential relevance of mitochondrial activities as therapeutic targets in MYC‐driven lymphoma was delineated further by transcriptome analysis across six patient‐derived DLBCL datasets, which revealed a close correlation between MYC‐ and OxPhos‐associated gene expression signatures [292]. In a previous study, transcriptional profiling led to the identification of a DLBCL subgroup characterized by high expression of OxPhos‐related genes [293]. DLBCL cell lines from this group were characterized by higher levels of TCA cycle activity and of the antioxidant glutathione, and accordingly by increased sensitivity to inhibitors of either fatty acid oxidation or glutathione biosynthesis [294]. In summary, an OxPhos gene signature is strongly correlated with oncogenic MYC activity and may point to therapeutically actionable processes in DLBCL.

Following from the above premises, we used IACS‐010759, a pharmacological inhibitor of electron transport chain (ETC) complex I [295], to show that oncogenic MYC sensitizes B‐cells to direct OxPhos inhibition. While IACS‐010759 was merely cytostatic in nontransformed B‐cells, it exerted a strong cytotoxic effect following ectopic activation of MYC in the same cells [292]. Mechanistically, MYC overexpression and IACS‐010759 treatment independently enhanced ROS production, causing lethal levels of oxidative stress and depletion of cellular glutathione [296], associated with activation of ISR signaling and intrinsic apoptosis [292]. In this context, the anti‐tumoral effects of IACS‐010759 could be reinforced by further exacerbation of oxidative stress, either by inhibiting NADPH biosynthesis through the pentose phosphate pathway, or by treating with pharmacological doses of ascorbate (vitamin C) [296]. Moreover, IACS‐010759 synergized not only with venetoclax to kill MYC/BCL2 DHL tumor cells *in vivo* and *in vitro*, as previously shown with tigecycline [289], but also with the MCL1 inhibitor S63845 against BCL2‐negative lymphoma cell lines [292]. Finally, IACS‐010759 also suppressed proliferation in Richter‐transformed CLL cells, which show high expression of OxPhos and MYC target genes [96]: it is tempting to speculate that venetoclax, S63845 or other BH3‐mimetic compounds may provide cooperative activity also in this context.

Taking a reverse approach, other authors identified increased OxPhos activity as a marker of venetoclax resistance in MM cells, and unraveled a similar synergy between IACS‐010759 and venetoclax [297]. Besides venetoclax, it was suggested that MM resistance to proteasome inhibition, a mainstay of MM therapy [298], might also be linked to increased OxPhos activity and glutathione levels [299]. Interestingly, advanced forms of MM expressed high levels of MYC and ETC subunits, and were suppressed by tigecycline *in vitro* and *in vivo* [300], pointing to mitochondrial inhibition as a possible strategy to overcome therapy resistance in MM. As already mentioned, ibrutinib resistance in MCL was also linked to increased expression of MYC and OxPhos gene signatures and, once again, these ibrutinib‐resistant cells were sensitive to OxPhos inhibition with IACS‐010759 [101]. Finally, independent studies showed that maintenance of the leukemic stem cell compartment in CML required both MYC [301] and Oxphos activity, and was compromised by tigecycline treatment [302]: we surmise that MYC may also contribute to tigecycline sensitivity in CML stem cells.

We already mentioned the co‐amplification of genes encoding MCL1 an MYC in residual TNBC after chemotherapy [110]. It was subsequently shown that the two oncogenes collaborated to induce a chemoresistant stem‐like phenotype by promoting mitochondrial respiration, and that this phenotype depended upon HIF1α stabilization by mitochondrial ROS [303]. It remains to be addressed whether inhibiting OxPhos may suppress chemoresistance in this setting.

Finally, mutations of the tumor suppressor FBXW7, which among others drives MYC degradation, have been associated with resistance to various chemotherapeutic agents [304]. Proteome analysis of FBXW7 knockout cells revealed increased levels of known targets, including MYC and mitochondrial components [305]. While resistant to chemotherapeutic drugs, these cells were sensitive to tigecycline, an effect that could be reversed by depletion of MYC [305].

Taken together, the aforementioned studies suggest that high MYC activity not only contributes to therapy resistance, but concomitantly sensitizes cancer cells to OxPhos inhibitors. Targeting this MYC‐OxPhos axis emerges as a promising therapeutic concept against aggressive, refractory, and recurrent MYC‐associated malignancies.

### 4.5 The integrated stress response: a new player in the bypass of therapy resistance

Among the aforementioned studies, several involved ISR signaling in drug‐mediated killing of cancer cells [306] and this was verified with OxPhos inhibitors, including IACS‐010759 in MYC‐overexpressing lymphoma [292] and MM [297], as well as Tigecycline in FBXW7‐knockout cells [305]. Moreover, other drugs that killed FBXW7‐null cells, albeit with diverse primary mechanisms of action, were all shown to induce ISR signaling [305]. These observations are in apparent contrast with the known function of the ISR as one of those adaptive mechanisms induced by oncogenic stress that favor cancer cell survival and expansion, as shown in diverse tumor models [307, 308, 309, 310], including MYC‐driven lymphoma [311, 312]. This apparent paradox may be readily rationalized, however, based on the well‐documented dual role of the ISR in cell survival and death [284, 285]. Mechanistically, ISR signaling is engaged upon phosphorylation of the translation factor eIF2⍺ by any of four different kinases that are selectively activated by diverse stress stimuli, including oxidative stress, endoplasmic reticulum‐ and mitochondrion‐induced unfolded protein responses (UPR), RNA‐associated stresses, and others [284, 285, 306, 313]. Most noteworthy here, oncogenic MYC can activate the ISR/UPR through several of those stresses [310, 311, 312, 314, 315]. While repressing general translation, phospho‐eIF2⍺ promotes the translation of a subset of transcripts with short upstream open reading frames, including the mRNAs encoding ATF4 and other transcription factors (e.g., CHOP, ATF5). Together, these factors drive gene expression programs associated with protein homeostasis, autophagy, stress‐resistance, and cell survival. Under conditions of severe, unresolved stress, such as those induced by IACS‐010759 treatment [292], the ISR may also promote apoptosis by inducing the expression of pro‐apoptotic BCL2‐family proteins [284, 285, 306, 313].

Altogether, while fulfilling a cytoprotective action when activated at moderate levels – as observed with oncogenic MYC – ISR signaling can be exacerbated by a large repertoire of targeted drugs, and thus to exert potent cell‐killing activity, which can overcome resistance to classical therapeutic regimens [305].

## 5 Conclusions

Over four decades of intense research activity have led to an advanced understanding of the physiological and pathological functions of MYC, uncovering it as a key pan‐cancer inducer of malignant phenotypes. Compelling clinical evidence associating MYC with resistance to multiple drug classes further points to this oncogene as a prime therapeutic target in oncology. While no MYC‐inhibitory drug has yet been approved for clinical use, recent progress in this area warrants advanced assessment of promising candidates. In a complementary approach, cell‐intrinsic and systemic dependencies elicited by oncogenic MYC provide new opportunities to exploit synthetic lethality toward the development of novel targeted interventions. Altogether, we are witnessing the emergence of diverse rationally designed strategies (Fig. 1), which shall significantly expand our toolbox to tackle oncogenic MYC and improve cancer patient outcomes.

## Conflict of interest

The authors declare no conflict of interest.

## Author contributions

GD and BA conceived and wrote the manuscript.

## Data accessibility

This review article included neither the production, nor the re‐analysis of original data.
